# Supplementary material for: Ensemble cryo-EM uncovers inchworm-like translocation of a viral IRES through the ribosome
Source: eLife. 2016 May 9;5:e14874. doi: 10.7554/eLife.14874 (PMC4896748; doi:10.7554/eLife.14874)
Supplement: Figure 1—source data 1. — DOI: http://dx.doi.org/10.7554/eLife.14874.003 [file elife-14874-fig1-data1.pdf]

Supplement to Figure 1; (1-S4). Structure refinement statistics for Structures I, II, III, IV, V

**Structures**	**Resolution (FSC=0.143)**	**Real-space R-factor**	**RMSD * Bond lengths (Å)**	**RMSD Angles (°)**
I	4.0	0.255	0.017	1.2
II	3.5	0.197	0.014	1.1
III	4.2	0.271	0.02	1.2
IV	4.0	0.235	0.016	1.1
V	3.95	0.225	0.016	1.1

* RMS (root-mean-square) deviations from ideal covalent bond lengths and angles (Engh and Huber, 1991).
