# Supplementary material for: Ensemble cryo-EM uncovers inchworm-like translocation of a viral IRES through the ribosome
Source: eLife. 2016 May 9;5:e14874. doi: 10.7554/eLife.14874 (PMC4896748; doi:10.7554/eLife.14874)
Supplement: Figure 2—source data 1. — DOI: http://dx.doi.org/10.7554/eLife.14874.008 [file elife-14874-fig2-data1.pdf]

Supplement to Figure 2; (2-S1). Measurements for conformations and positions in Structures I through V.

Structure<br>PDB ID<br>Reference	80S•IRES<br>3J6Y (3J6X)<br>Koh *et al.* (2014)	80S•IRES•eEF2•GDP•sordarin<br>This work					80S•1tRNA<br>3J77	80S•2tRNAs<br>3J78
		I	II	III	IV	V	Svidritskiy *et al.* (2014)	
**Position of PKI on the 40S**	**A site**	**Between A and P sites**				**P site**	**Reference Structures (no IRES)**	
**IRES translocation state**	INITIATION (no eEF2)	PRE-translocation (+ eEF2)	MID-translocation (+ eEF2)	MID-translocation (+ eEF2)	MID-translocation (+ eEF2)	POST-translocation (+ eEF2)	tRNA PRE-translocation (no eEF2, with one P/E tRNA)	tRNA POST-translocation (no eEF2, with two tRNAs)
**Intersubunit rotation**<br>40S rotation relative to 60S	Mid-rotated 3.7° (7.2°)	Fully rotated 9.6°	Mid-rotated 4.6°	Mid-rotated 4.5°	Almost non-rotated 1.3°	Least rotated 0.5°	**Fully rotated 40S 11.0°**	**Non-rotated 40S 0°**
**40S Head swivel**<br>Beak rotation toward P-site	Mid - swiveled 10.1° (9.7°)	Mid-swiveled 11.6°	Most swiveled 17.1°	Most swiveled 17.1°	Mid - swiveled 13.8°	Least swiveled 1.1°	**Non-swiveled 40S-Head 2.3°**	**Non-swiveled 40S-Head 0°**
**IRES conformation**<br>Distance between PKI and SL4, at phosphates U6913 – A6848	Extended 47 Å (47 Å)	Partially bent 36 Å	Partially bent 34 Å	Highly bent 28 Å	Highly bent 26 Å	Extended 44 Å	-	-
**Distances**	**PKI and eEF2 position relative to the P-site stacking platform (U1191 and C1637)**							
**PKI to Body P site**<br>G6905, C4 – C1637, N1	16 Å (18 Å)	13 Å	7 Å	6 Å	5 Å	4 Å	-	-
**PKI to Head P site**<br>G6905, O4' – U1191, O4'	23 Å (21 Å)	19 Å	18 Å	11 Å	8 Å	4 Å	-	-
**eEF2 tip to Body P site**<br>H657, Cα – C1637, O4'	-	35 Å	27 Å	27 Å	26 Å	25 Å	-	-
**eEF2 tip to Head P site**<br>H657, Cα – U1191, O4'	-	41 Å	38 Å	37 Å	32 Å	24 Å	-	-
**Distances**	**eEF2 position relative to A site (post-translocation eEF2 in Structure V)**							
**Domain IV**<br>H657, Cα – H657, Cα (V)	-	11 Å	4 Å	5 Å	2 Å	0 Å (Reference)	-	-
**Domain I**<br>E265, Cα – E265, Cα (V)	-	18 Å	9 Å	8 Å	2 Å	0 Å (Reference)	-	-
